# Advances in Biomimetics: The Power of Diversity

**DOI:** 10.3390/biomimetics10010054

**Published:** 2025-01-15

**Authors:** Stanislav N. Gorb, Giuseppe Carbone, Thomas Speck, Peter Fratzl

**Affiliations:** 1 Department of Functional Morphology and Biomechanics, Zoological Institute, Kiel University, 24118 Kiel, Germany; 2 Dipartimento di Meccanica-Matematica-Management DMMM, Campus, Via Orabona 4, 70125 Bari, Italy; giuseppe.carbone@poliba.it; 3 Plant Biomechanics, Botanic Garden, Faculty of Biology, University of Freiburg, Schänzlestraße 1, D-79104 Freiburg, Germany; thomas.speck@biologie.uni-freiburg.de; 4 Cluster of Excellence livMatS @ FIT, Georges-Köhler-Allee 105, D-79110 Freiburg, Germany; 5 Max Planck Institute of Colloids and Interfaces, Department of Biomaterials, Research Campus Golm, Am Mühlenberg 1, 14476 Potsdam, Germany; peter.fratzl@mpikg.mpg.de

Biomimetics research on living systems attempts to transfer their properties and functions to engineering applications. Biological materials, structures, and processes are predominantly based on the combination of various effects at different scales: from the nano- through to the micro-, meso-, and, finally, the macroscale. This Special Issue is devoted to the latest advances in biomimetics in all its subfields: (1) materials and structures; (2) designs, constructions, and devices; (3) surfaces and interfaces; (4) locomotion and bioinspired robotics; and (5) development of biomimetic methodology. The Special Issue contains papers from biological fields focusing on the proper identification of the underlying structure and functional principles in nature, which explains the observed properties of biological systems. Manuscripts that apply these to modern technologies have been included as well.

Two important review papers are included: the first is on scaffold-based blood vessel tissue engineering by Elisabetta Rosellini and coworkers (2024) [Contribution 1] and the second is on metachronal motion of biological and artificial cilia by Zhiwei Cui et al. (2024) [Contribution 2]. The first review provides the state of the art of vascular tissue engineering, specifically focusing on scaffolds designed by following a biomimetic approach. By mimicking native vessel properties, and particularly the complexly layered structure of the vascular wall, tissue-engineered scaffolds demonstrate improved long-term patency and promising clinical results. Moreover, modern biomimetic research is focused on enhancing the use of innovative scaffold materials, surface functionalization strategies, and the use of bioreactors mimicking the physiological microenvironment. Finally, this paper provides an overview of the latest advancements and future directions of vascular tissue engineering, with emphasis on the use of biomimetics to generate systems capable of reproducing the structure–function relationships present in the arterial wall (Rosellini et al., 2024) [Contribution 1]. The second review focuses on biological and artificial cilia. Biological cilia are slender, hair-like cell protrusions present in numerous organisms from protozoans to humans. They generate fluid flow, participate in locomotion, and support feeding. Their coordinated beating resulting in wavelike motions (so-called metachrony) supports flow generation and mucus transport. Researchers have meanwhile undertaken numerous attempts to fabricate artificial cilia and develop innovative approaches to mimic the metachronal motion observed in nature. In this review, different types of metachronal motion generated by both biological and artificial cilia are analyzed (pneumatically, photonically, electrically, and magnetically driven). Numerous possible applications of artificial cilia are proposed, and potential future directions within this field are suggested (Cui et al., 2024) [Contribution 2].

The simulation and experimental study by Fang et al. (2024) [Contribution 3] is focused on the role of tail dynamics on the climbing performance of gecko-inspired robots. They claim that the majority of research that has focused on the adhesion mechanisms of geckos has ignored that the tail plays a critical role in maintaining balance and stability during locomotion on the wall and ceiling. The authors systematically explore the role of tails on the climbing performance of gecko-inspired robots using both simulation and experimental approaches and developed a simulation system that predicts the robot’s contact failures (Fang et al., 2024) [Contribution 3]. Their work provides an important step for further optimizing climbing robot performance.

The paper by Casagualda et al. (2024) [Contribution 4] reports on novel mussel-inspired multifunctional polyethylene glycol nanoparticle interfaces. The authors developed a bioinspired catechol-based strategy to obtain biocompatible and multifunctional coatings based on a previously developed polymerization methodology under mild oxidative conditions. Two types of nanoparticles were made: (1) mesoporous-silica-based nanoparticles and (2) magnetite-based nanoparticles. The authors have not only demonstrated the feasibility of their approach in obtaining coatings by using different types of nanoparticles but also incorporated fluorescein functionalities into their coatings that confer biocompatibility and excellent cell internalization and can be used for the imaging and tracking of nanoparticles in various biological and medical applications (Casagualda et al., 2024) [Contribution 4].

Sponges are well recognized as an archive of multi-scaled skeletal constructs with superficial micro-ornamentation patterned by biopolymers (Ehrlich et al., 2024) [Contribution 5]. In their study, Ehrlich et al. (2024) [Contribution 5] desilicified spicules and skeletal frameworks of some selected sponge species using 10% HF and obtained isolated axial filaments consisting of F-actins. These F-actins, called silactins, are presumably pattern drivers in skeletal constructs of sponges, and their understanding may open (1) the way to the fundamental understanding of the skeletogenesis of sponges and (2) the biomimetic potential towards the synthesis of poriferan biosilica patterned by silactins (Ehrlich et al., 2024) [Contribution 5].

Winand et al. (2024) [Contribution 6] have developed a robotic gripper (TriTrap gripping device) inspired by insect tarsal chains. In their paper, they highlight the potential of the tarsal chain principle. Just like its biological counterpart, the TriTrap utilizes strongly underactuated digits that function using morphological encoding and passive conformation. This results in a gripper that is versatile, robust, and low cost. The gripping performance of the TriTrap is demonstrated on a variety of 3D objects of different sizes, weights, and shapes (Winand et al., 2024) [Contribution 6]. The future potential of the insect tarsus principle in robotics in general is discussed.

The paper by Chen et al. (2024) [Contribution 7] is devoted to the liquid interaction with *Heliamphora minor* mimicking mesoscopic trichome arrays. It is well known that driven by the phenomenon of capillarity, a liquid can spread on rough lyophilic surfaces (capillary wicking). In this paper, the authors were inspired by the pitcher plant *H. minor*, which is capable of spreading water on its inside wall covered by anisotropically oriented trichomes. In the next step, the authors used a 3D printing technique to fabricate similar surface structures and investigated their capillary wicking capability. They also demonstrated the mass transportation on this biomimetic surface and suggested potential applications of their development in the design of low-cost, high-flux open fluidic devices (Chen et al., 2024) [Contribution 7].

The paper by Saint-Sardos et al. (2024) [Contribution 8] discusses the importance of the taxonomy-driven exploration of biodiversity for potential bioinspired innovations. The recent problem of searching for bioinspiration is due to the presentation of open-access biodiversity databases that are often adapted to life scientists rather than to bioinspired designers and engineers. In this paper, the authors suggested a new tool called “Bioinspire-Explore” capable of navigating biodiversity data to uncover biological systems of interest for different areas of bioinspired engineering. This tool allows for searching biological models using a taxonomy approach and provides information on the taxon’s position, its distribution, and its ecological niche (Saint-Sardos et al., 2024) [Contribution 8]. This novel tool has strong potential for biologists, but especially for bioinspired designers in gathering quick and reliable information about the organisms and their features that are potentially important for biomimetics.

Wang et al. (2024) [Contribution 9] reported on the target-following control of a biomimetic autonomous system inspired by the fish swimming in a group with improved hydrodynamic efficiency. In the established model, the follower robotic fish keeps a certain distance and orientation from the leader fish. Then a nonlinear predictive controller was designed that can be selected for the follower together with the predictive reinforcement learning (Wang et al., 2024) [Contribution 9]. After extensive simulations, the authors demonstrated the effectiveness of the cooperative control for underwater swarm locomotion.

The mechanisms that coordinate leg movement patterns are especially complex in organisms moving at the water–air interface. Meshkani et al. (2023) [Contribution 10] assumed the presence of compensatory factors, which are involved in the maintenance of the water strider posture after the amputation of individual legs. The authors studied load distribution among the legs and analyzed the effects of leg amputation on the locomotory behavior. A stable posture was quickly recovered by animals by using leg position modifications and load redistribution to the remaining legs. These experiments may assist the bioinspired design of robust aquatic robots (Meshkani et al., 2023) [Contribution 10].

Thanks to the theoretical, experimental, and review contributions provided by researchers in the fields of biology, physics, material science, engineering, this Special Issue of *Biomimetics* will certainly find its broad readership among all researchers who are engaged in this fast-growing field of science.

